# 6-Gingerol Inhibits Growth of Colon Cancer Cell LoVo via Induction of G2/M Arrest

**DOI:** 10.1155/2012/326096

**Published:** 2012-06-07

**Authors:** Ching-Bin Lin, Chun-Che Lin, Gregory J. Tsay

**Affiliations:** ^1^Division of Hepatology and Gastroenterology, Department of Internal Medicine, Chung Shan Medical University Hospital, Taichung 402, Taiwan; ^2^Institute of Microbiology and Immunology, Chung Shan Medical University, Taichung 402, Taiwan; ^3^Institute of Medicine, Chung Shan Medical University, Taichung 402, Taiwan; ^4^Department of Medicine, Chung Shan Medical University (CSMU), Taichung 402, Taiwan

## Abstract

6-Gingerol, a natural component of ginger, has been widely reported to possess antiinflammatory and antitumorigenic activities. Despite its potential efficacy against cancer, the anti-tumor mechanisms of 6-gingerol are complicated and remain sketchy. In the present study, we aimed to investigate the anti-tumor effects of 6-gingerol on colon cancer cells. Our results revealed that 6-gingerol treatment significantly reduced the cell viability of human colon cancer cell, LoVo, in a dose-dependent manner. Further flow cytometric analysis showed that 6-gingerol induced significant G2/M phase arrest and had slight influence on sub-G1 phase in LoVo cells. Therefore, levels of cyclins, cyclin-dependent kinases (CDKs), and their regulatory proteins involved in S-G2/M transition were investigated. Our findings revealed that levels of cyclin A, cyclin B1, and CDK1 were diminished; in contrast, levels of the negative cell cycle regulators p27^Kip1^ and p21^Cip1^ were increased in response to 6-gingerol treatment. In addition, 6-gingerol treatment elevated intracellular reactive oxygen species (ROS) and phosphorylation level of p53. These findings indicate that exposure of 6-gingerol may induce intracellular ROS and upregulate p53, p27^Kip1^, and p21^Cip1^ levels leading to consequent decrease of CDK1, cyclin A, and cyclin B1 as result of cell cycle arrest in LoVo cells. It would be suggested that 6-gingerol should be beneficial to treatment of colon cancer.

## 1. Introduction

Colorectal cancer (CRC) is one of the most prevalent cancers with high mortality in the western world and Taiwan [1]. CRC is inclined to evolve into invasive cancer from adenomatous polyps through mutations in various genes [2]. Although early diagnosis improves patients' clinical outcomes, 5-year survival rate of patients diagnosed with CRC is poor. Current therapeutic regimens for CRC constitute predominantly of surgical procedures and chemotherapy [3, 4]. Despite improvements in the prognosis of CRC patients receiving appropriate clinical modularity, resistance to advanced therapy does occur in many patients suffering from incomplete eradication of malignant cells and metastasis.

Of various phytochemicals showing various biochemical and pharmacologic activities, 6-gingerol, a major pharmacologically active component of ginger, has been reported to exhibit antioxidant and anti-inflammatory properties and exert substantial anticarcinogenic and antimutagenic activities [5]. Mounting evidence suggests that 6-gingerol is effective in suppressing the transformation, hyperproliferation, and inflammatory processes that initiate and promote carcinogenesis, as well as the later steps of carcinogenesis, namely, angiogenesis and metastasis [6–10]. Despite awareness to its activity against several human cancers, the exact molecular mechanism underlying anti-tumoral effects of 6-gingerol remains sketchy.

Accumulating evidence suggests that induction of reactive oxygen species (ROS) by phytochemicals are critically involved in their anti-tumoral activity [11, 12]. Increase of intracellular ROS usually leads to DNA damage, and the subsequent phosphorylation of p53 contributes to cell cycle arrest and further apoptosis of cancer cell. The role of cell cycle mediators in cancer development is now well documented. Critical genes responsible for cell cycle regulation as checkpoints have been demonstrated to be lost and/or aberrant in a variety of cancers in human [13]. Cell cycle is under sophisticated regulation through the interactions of different cyclins with their specific kinases, cyclin-dependent kinases (CDKs) [14]. Two classes of CDK inhibitors, inhibitors of CDK4 (INK4) and kinase inhibitor proteins (KIPs), have been reported to negatively modulate the activity of CDKs. The latter include p21^Cip1^ [15], p27^Kip1^ [16], and p57^Kip2^ [17, 18]. It has been reported that overexpression of p21^Cip1^ leads to inhibited proliferation of mammalian cells and inactivation of all cyclin-CDK complexes, indicating that it is a universal cyclin-CDK inhibitor [19]. p27^Kip1^, a negative regulator of protein kinases, interacts with cyclin E-CDK2 and cyclin A-CDK2 which drive cells into the S phase of the cell division cycle [20]. Moreover, p27^Kip1^ has been reported to play important roles in G2/M checkpoint as tumor suppressor [21].

In the present study, we focused on the mechanism underlying anticancer effects of 6-gingerol on colon cancer with emphasis on cell viability alteration and cell cycle disruption. To investigate the alteration of cell viability and cell cycle distribution induced by 6-gingerol, MTT assay and flow cytometric analysis were performed. Expression level of important cell cycle regulators was determined by immunoblotting. Intracellular ROS was determined by using spectrofluorometrical analysis.

## 2. Materials and Methods

### 2.1. Materials

 6-gingerol, 2-propanol, 3-(4,5-Dimethylthiazol-2-yl)-2,5-diphenyltetrazolium bromide (MTT), 1-butanol, dimethyl sulfoxide (DMSO), 2′,7′-dichlorofluorescein diacetate (DCF-DA), deoxycholic acid, dithiothreitol, EDTA, glycerol, Igepal CA-630, phenylmethylsulfonyl fluoride (PMSF), sodium chloride (NaCl), potassium chloride (KCl), sodium dodecyl sulfate (SDS), sodium phosphate, Tris-HCl, and trypsin/EDTA were purchased from Sigma (St. Louis, MO, USA). Antibodies against cyclin A, cyclin B1, cyclin D1, cyclin E, CDK1, p53, p21^Cip1^, p27^ Kip1^, and *β*-actin were purchased from Santa Cruz Biotechnology (Santa Cruz, CA, USA). Peroxidase-conjugated antibodies against mouse IgG or rabbit IgG were purchased from Cell Signaling Technology (Beverly, MA, USA).

### 2.2. Cell Culture

 Colon cancer cell line LoVo was obtained from the American Type Culture Collection (ATCC; Rockville, MD, USA) and maintained in Dulbecco's modified Eagle's medium (DMEM) supplemented with 10% v/v fetal bovine serum, 1% nonessential amino acid, 1% L-glutamine (Gibco BRL, Gaithersburg, MD, USA), and 100 *μ*g/mL penicillin/streptomycin (Sigma) at 37°C in a humidified atmosphere with 5% CO_2_. Cells were seeded in 10 cm Petri dishes at an initial density of 2 × 10^5^ cells/mL and grown to approximately 80% confluence. Then, the cells were collected for the subsequent analyses including cell viability, flow cytometric analysis, and immunoblotting analysis.

For 6-gingerol treatments, cells were starved for 24 hours (h) in serum-free DMEM and then incubated with 6-gingerol at a series of concentrations in serum-free DMEM (1, 5, 10, and 15 *μ*g/mL) for 24 h or 48 h.

### 2.3. Cell Viability Assay

 Cell viability was determined by MTT assay as previously described [20]. Briefly, cells were seeded at a density of 4 × 10^4^ cells/well in a 24-well plate and cultured with serum-free DMEM for 16 h. Then, the cells were treated with serial concentrations of 6-gingerol (0, 5, 10, and 15 *μ*g/mL) for 24 h or 48 h. Treatment at each concentration was performed in triplicate. After treatments, the medium was aspirated and cells were washed with PBS. Cells were subsequently incubated with MTT solution (5 mg/mL) for 4 h. The supernatant was removed, and formazan was solubilized in isopropanol and measured spectrophotometrically at 563 nm. The percentage of viable cells was estimated in comparison with untreated cells.

### 2.4. Determination of Cell Cycle Distribution

 Cell cycle distribution was analyzed by flow cytometry. After 6-gingerol treatment, cells were collected, fixed with 1 mL of ice-cold 70% ethanol, incubated at −20°C for at least 24 h, and centrifuged at 380 ×g for 5 min at room temperature. Cell pellets were treated with l mL of cold staining solution containing 20 *μ*g/mL propidium iodide (PI), 20 *μ*g/mL RNase A, and 1% Triton X-100 and incubated for 15 min in dark at room temperature. Subsequently, the samples were analyzed in a FACS Calibur system (version 2.0, BD Biosciences, Franklin Lakes, NJ, USA) using Cell Quest software. Results were representative of at least three independent experiments.

### 2.5. Protein Extraction

 After 6-gingerol treatments, cells were trypsinized and homogenized in ice-cold lysis buffer (50 mM Tris-HCl, pH 7.5, 150 mM NaCl, 0.1% (v/v) Igepal CA-630, 0.5% (w/v) sodium deoxycholate, 0.1% (w/v) SDS, 1 mM dithiothreitol, 0.1 mM EDTA, and 1 mM PMSF). After sonication at 4°C for 30 min, the homogenate was centrifuged at 14,000 ×g for 10 min, and then the supernatant was transferred into a new 1.5 mL eppendorf and stored at −70°C for subsequent analysis. Protein concentration was quantitated by the Bradford method (protein assay reagent; Bio-Rad Laboratory, Hercules, CA, USA) according to the manufacturer's instruction.

### 2.6. Immunoblotting

 Crude proteins (30 *μ*g of protein) were subjected to a 12.5% SDS-polyacrylamide gel and transferred onto a nitrocellulose membrane as previously described [21]. The blot was subsequently incubated with 5% nonfat milk in PBS for 1 h, probed with a primary antibody against cyclin A, cyclin B1, CDK1, p21^Cip1^, p27^Kip1^, p53, or *β*-actin for 2 h and then reacted with an appropriate peroxidase-conjugated secondary antibody for 1 h. All incubations were carried out at 30°C, and intensive PBS washing was performed between incubations. After the final PBS wash, the signal was developed by ECL chemiluminescence, and the relative photographic density was quantitated by image analysis system (Fuji Film, Tokyo, Japan).

### 2.7. Determination of Intracellular Reactive Oxygen Species (ROS)

 Production of ROS was determined by spectrofluorometrical method using 2′,7′-dihydrodichlorofluorescein diacetate (DCFH-DA) assay with modification [22]. DCFH-DA diffuses through the cell membrane and is enzymatically hydrolyzed by intracellular esterases to the nonfluorescent DCFH, which can be rapidly oxidized to the highly fluorescent DCF, the fluorescent product, in the presence of ROS. After exposure to LPS and PFE, DCFH-DA was added to the culture plates at a final concentration of 5 *μ*M and incubated for 40 min at 37°C in darkness. DCF fluorescence intensity was detected with emission wavelength at 530 nm and excitation wavelength at 485 nm using a SpectraMax Plus microplate reader (Molecular Devices Corporation, Sunnyvale, CA, USA). The values were expressed as the mean absorbance normalized to the ratio of control value.

### 2.8. Statistical Analysis

 Data were expressed as mean ± standard deviation (SD) of the three independent experiments. Statistical significance analysis was determined by using 1-way ANOVA followed by Dunnett for multiple comparisons with the control. The differences were considered significant for *P* values less than 0.05.

## 3. Results

### 3.1. 6-Gingerol Inhibited the Cell Viability of LoVo Cells

**Figure 1:**
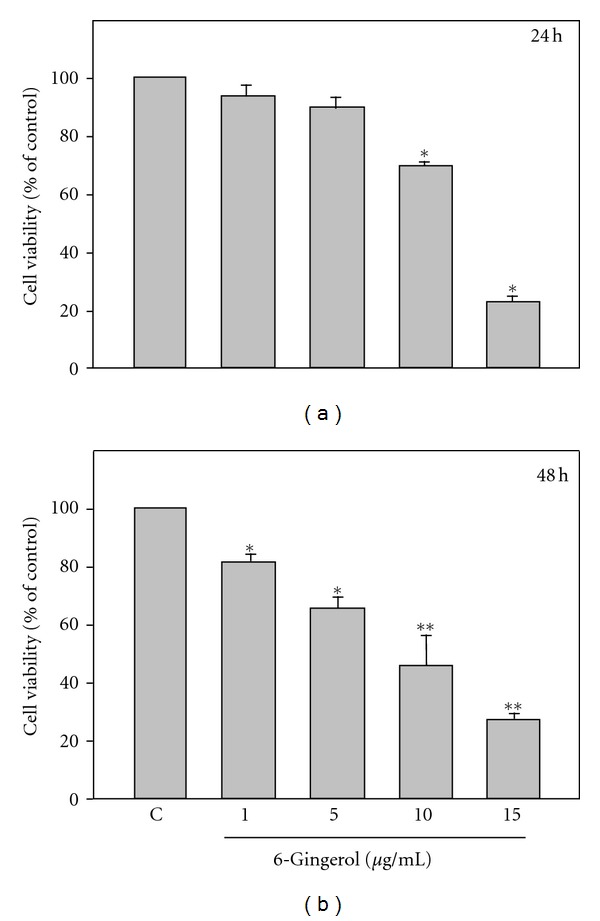
6-Gingerol inhibited the cell viability of LoVo cells. Cells were treated with indicated concentration of 6-gingerol for 24 h or 48 h, and the cell viability was analyzed by MTT assay. Data were shown as the means ± SD. Three independent experiments were performed for statistical analysis. *P < 0.05 and **P < 0.005 as compared to control (C).

 To examine the inhibitory effects of 6-gingerol on colon cancer cells, LoVo cells were treated with a serial concentration of 6-gingerol (1, 5, 10, and 15 *μ*g/mL) for 24 or 48 h, respectively, and then cell viability of LoVo cells was determined. As shown in Figure 1, the cell viability in presence of 6-gingerol was found decreased in association with the concentration of 6-gingerol in a dose-dependent fashion. 6-Gingerol treatments at concentrations of 10 and 15 *μ*g/mL significantly decreased cell viability to 68.7 ± 4.3% and 24.6 ± 2.1% of control for 24 h and to 40.4 ± 1.4% and 24.5 ± 1.4% of control for 48 h, respectively (*P* < 0.05 as compared to control).

### 3.2. 6-Gingerol Induced G2/M Phase Arrest but Not Apoptosis in LoVo Cells

**Figure 2:**
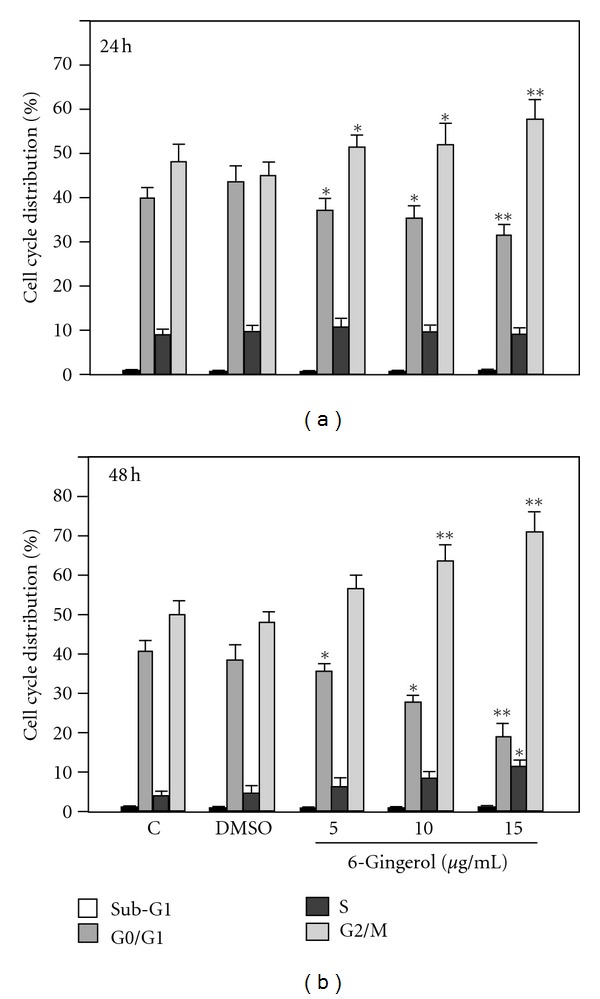
6-Gingerol induced G2/M phase arrest of LoVo cells. Cells were treated with indicated concentration of 6-gingerol for 24 h or 48 h, and the percentages of various cell cycle phases, including sub-G1, G0/G1, S, and G2/M, were analyzed and quantitated by flow cytometry. Data were shown as the means ± SD. Three independent experiments were performed for statistical analysis. *P < 0.05 and **P < 0.005 as compared to corresponding control.

 As a significant suppression of cell viability of LoVo occurred after 6-gingerol treatments resulted, cell cycle distribution of 6-gingerol-treated LoVo cell was consequently analyzed and quantitated using flow cytometry. As shown in Figure 2, percentages of cells in sub-G1 phase, ranging from 1.36 ± 0.23% to 2.58 ± 0.36%, were not significantly influenced by the treatments of 6-gingerol for 24 h. However, an increase in population of cells in G2/M phase after the treatment was observed in a dose-dependent manner, ranging from 45.7 ± 3.6% to 58.8 ± 5.4%, (5, 10 and 15 *μ*g/mL, *P* < 0.05). Additionally, a number of G0/G1 phase cells, ranging from 43.8 ± 2.9% to 33.7 ± 3.2%, were significantly decreased with the concentration of 6-gingerol. The similar change in population of G2/M phase and G0/G1 phase was also found in LoVo cells treated with the serial concentrations of 6-gingerol for 48 h. These results revealed that 6-gingerol treatments increased the ratios of G2/M phase but decreased G0/G1 phase of LoVo cells in a dose-dependent manner. Moreover, 15 *μ*g/mL 6-gingerol treatment resulted in an 1.29-fold increase in number of cells in G2/M phase compared with that after DMSO treatment. Amongst 4 phases of cell cycle, G2/M phase arrest of LoVo cells was significant in response to 6-gingerol treatment.

**Figure 3:**
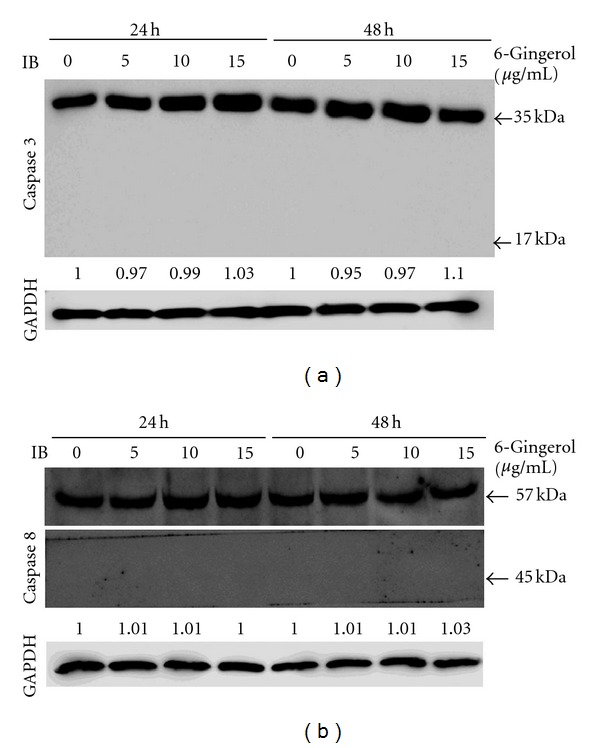
Effects of 6-gingerol on activation of caspase 3 and caspase 8 of LoVo cells. Cells were treated with indicated concentration of 6-gingerol for 24 h or 48 h, and then the cell lysates were subjected to immunoblot for detection of caspase 3 and caspase 8. Protein levels were relatively quantitated by densitometric analysis using GAPDH as control.

As a slight change in percentage of sub-G1 phase of 6-gingerol-treated LoVo cells was observed, a further experiment was performed to investigate the involvement of apoptosis in inhibited viability of LoVo cells upon exposure to 6-gingerol. Caspase 3, and 8 that are situated at pivotal junction in apoptosis pathway were monitored after 6-gingerol treatment. No significant change in the level of precursor form and activated form of caspase 3 was observed in response to 6-gingerol treatments (5, 10, and 15 *μ*g/mL) as well as caspase 8 (Figure 3).

### 3.3. 6-Gingerol Diminished Levels of CDK1, Cyclin A, and Cyclin B1 in LoVo Cells

**Figure 4:**
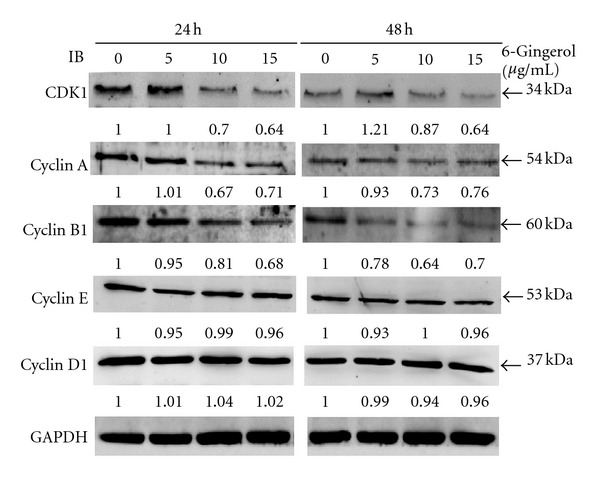
Effects of 6-gingerol on CDK1 and cyclins of LoVo cells. Cells were treated with indicated concentration of 6-gingerol for 24 h or 48 h, and then the cell lysates were subjected to immunoblot for detection of indicated proteins. Protein levels were relatively quantitated by densitometric analysis using GAPDH as control.

 Having observed 6-gingerol-induced G2/M phase arrest, the effects of 6-gingerol treatments on cell cycle progress of LoVo cells were further investigated. Levels of important cell cycle mediators, including CDK1, cyclin A, cyclin B1, cyclin D1, and cyclin E, were determined by immunoblotting and relatively quantitated by densitometric analysis. Our results showed that 6-gingerol treatments (5, 10, and 15 *μ*g/mL) dose-dependently decreased the levels of CDK1, cyclin A, and cyclin B1 but slightly affected the levels of cyclin D1 and cyclin E (Figure 4). With the 6-gingerol treatment at concentration of 15 *μ*g/mL for 24 h, the levels of CDK1, cyclin A, and cyclin B1 were reduced to 64%, 71%, and 68% of control, respectively, by densitometric quantitation (Figure 4).

### 3.4. 6-Gingerol Increased Levels of p21^Cip1^ and P27^Kip1^ in LoVo Cells

**Figure 5:**
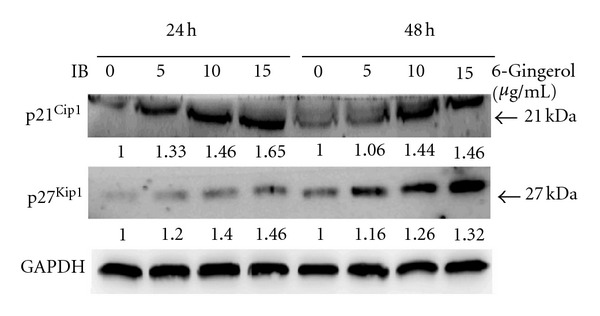
Effects of 6-gingerol on p21^Cip1^ and p27^Kip1^ of LoVo cells. Cells were treated with indicated concentration of 6-gingerol for 24 h or 48 h, and then the cell lysates were subjected to immunoblot for detection of p21^Cip1^ and p27^Kip1^. Protein levels were relatively quantitated by densitometric analysis using GAPDH as control.

 Observing diminished levels of CDK1, cyclin A, and cyclin B1 upon 6-gingerol treatments, we further investigated the effects of 6-gingerol treatments on cell cycle regulators, p21^Cip1^ and p27^Kip1^. As shown in Figure 5, 6-gingerol treatments (24 h) dose-dependently increased levels of p21^Cip1^ and p27^Kip1^ up to 1.65- and 1.46-fold, respectively, compared to that of control. The trend of increase in p21^Cip1^ and p27^Kip1^ level was continuous in LoVo cells for further 24 h. These findings revealed that 6-gingerol treatments significantly induced both of negative cell cycle regulators p21^Cip1^ and p27^Kip1^.

### 3.5. 6-Gingerol Elevated p53 Level and Intracellular ROS in LoVo Cells

**Figure 6:**
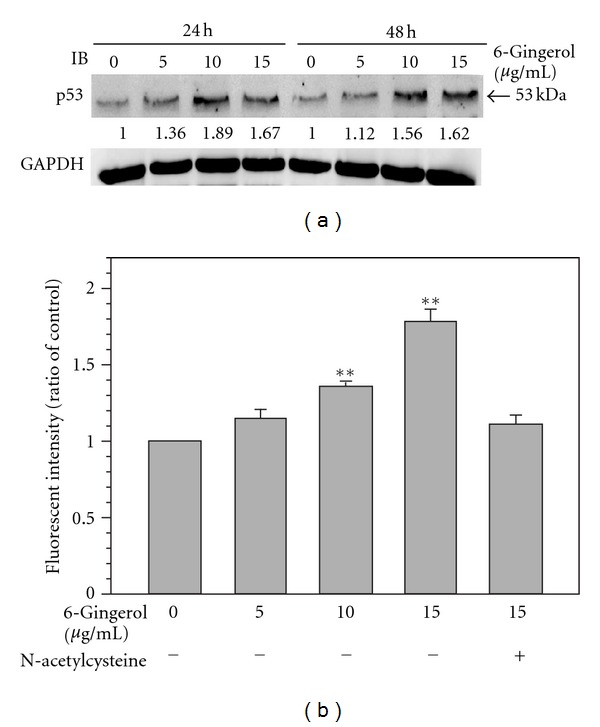
Effects of 6-gingerol on p53 and intracellular ROS of LoVo cells. (a) Cells were treated with indicated concentration of 6-gingerol for 24 h or 48 h, and then the cell lysates were subjected to immunoblot for detection of p53. Protein levels were relatively quantitated by densitometric analysis using GAPDH as control. (b) Cells were treated with indicated concentration of 6-gingerol for 24 h, and the intracellular ROS was determined as described in the Section 2.

 Basing on that 6-gingerol treatment elevated negative cell cycle regulator p21^Cip1^, the upstream regulator of p21^Cip1^, p53 was further investigated. As shown in Figure 6(a), 6-gingerol treatments (24 h) elevated level of p53 up to 1.89-fold as compared to that of control. The trend of increase in p53 level was continuous in LoVo cells for further 24 h. These findings revealed that 6-gingerol treatments significantly induced the important cell cycle regulator p53 in LoVo cells.

ROS has been reported to play pivotal roles in phytochemical-induced cell cycle arrest and apoptosis [23, 24]. Therefore, whether 6-gingerol induced ROS production in LoVo cells was also analyzed. As shown in Figure 6(b), 6-gingerol dose-dependently increased intracellular ROS up to 1.89-fold as compared to the control, and the increase of ROS was diminished by NAC pretreatment. These results showed that 6-gingerol significantly increased level of p53 as well as elevated intracellular ROS in LoVo cell.

## 4. Discussion

Previous studies have shown that treatment of 200 *μ*M 6-gingerol induced G1 phase arrest and apoptosis in several human colorectal cancer cells, including HCT-116, SW480, HT-29, LoVo, and Caco-2 [25]. It is also reported that 6-gingerol (60 *μ*M) shows a weaker effect on induction of apoptosis of colorectal carcinoma COLO 205 than its analogue, 6-shogaol [26]. Similarly, our results demonstrate that a relative low concentration of 6-gingerol (up to 50 *μ*M) significantly suppresses the viability, induces G2/M phase arrest, but does not provoke apoptosis of LoVo cells. Therefore, it is suggested that low concentration of 6-gingerol tends to inhibit growth of LoVo cells through induction of cell cycle arrest instead of apoptosis.

Cyclin A2, an originally identified A-type cyclin, is ubiquitously expressed in mitotically dividing cells and is upregulated in a variety of cancers [27, 28]. In late G1 phase, cyclin A binds to CDK2 to promote transition to S phase and plays important roles in replication of DNA and centromere in S phase [29]. Another type of cyclin is discovered and coined as B-type cyclin of which the biological role is not fully understood; however, the B-type cyclins generally emerge during the G2-M phase transition of the cell cycle. During G2-M phase transition, cyclin B1 binds to CDK1 (cdc2) to form mitosis-promoting factor that facilitates the transition from G2 to M phase of the cell cycle [30]. Therefore, reduced levels of cyclin A and cyclin B1 attenuate the activation of both CDK1 and CDK2, consequently leading to the cell cycle arrest at S phase and G2/M phase. In consistency with the phenomenon, our flow cytometric analysis showed a significant increased percentage of G2/M phase in 6-gingerol-treated LoVo cells (Figure 2), suggesting that 6-gingerol may trigger the G2/M cell cycle arrest via downregulation of cyclin A, CDK2, cyclin B1, and CDK1.

Generally, the activity of cyclin-CDK complexes is regulated by two different families of proteins known as INK4 and CDK inhibitors [31]. However, the tight regulation of cell cycle progression is compromised in cancer cells, which consequently results in aberrant proliferation of cells [32]. In this regard, both INK4 and CDK inhibitor family members have been reported to lose their functions in various malignant cancers such as CRC, resulting in an uncontrolled cell cycle progression and cancer growth [33, 34]. Therefore, the molecular players such as cyclins, CDKs, and their inhibitors serve as potential targets to halt the uncontrolled proliferation [35, 36]. Specifically, it could be argued that the agents inducing the level and/or function of cell cycle inhibitory regulators (INK4 and Cip/Kip family members) might be useful in the control of various malignancies including CRC. In the present study, our results clearly showed an increase in the levels of p21^Cip1^ and p27^Kip1^ in presence of 6-gingerol in LoVo cells, which is in line with the observed G2/M phase arrest. Importantly, 6-gingerol caused a dose- and time-dependent increase in the levels of p27^Kip1^ in LoVo cells, which supports the finding of cell cycle arrest effect in S or G2/M phase in this cell line.

6-gingerol has been reported to exert its anti-tumoral activity via induction of ROS which is also known to trigger activation of p53 and the consequent cell cycle arrest and apoptosis [37]. Our results also showed that 6-gingerol significantly increased intracellular ROS as well as the critical cell cycle regulator p53 in LoVo cells. These findings indicate that 6-gingerol increased p53 level may attribute to induction of ROS. In conclusion, it could be suggested that 6-gingerol induces ROS production and p53 activation as well as inhibits the degradation of p27^Kip1^ and p21^ Cip1^ in LoVo cells, by a mechanism yet to be established, which induces the cell cycle arrest at S and G2/M phases.
